# Profile of people seeking health services during Pope Francis’ visit to Mozambique, 2019

**DOI:** 10.11604/pamj.2020.35.95.21611

**Published:** 2020-04-01

**Authors:** Hélio Inácio Elias, Eduardo Mangue Chicanequisso, Beatriz Nhantumbo, Judite Monteiro Braga, Lorna Gurjal, Maiumuna Luis, Tomas Dimas, Cynthia Semá Baltazar, Erika Valeska Rossetto

**Affiliations:** 1 Mozambique Field Epidemiology Training Program, Instituto Nacional de Sade, Maputo, Mozambique; 2 Instituto Nacional de Saúde, Maputo, Mozambique; 3 Epidemiology Department, National Public Health Directorate, Ministry of Health, Maputo, Mozambique; 4 MassGenics assigned to Mozambique Centers for Disease Control and Prevention, Maputo, Mozambique

**Keywords:** Mozambique, epidemiological, health surveillance, mass gathering, Pope Francis

## Abstract

Pope Francis visited Mozambique from September 4-6, 2019. During the visit, a real-time surveillance system for mass gathering events was implemented in all places where people gathered in Maputo City for early detection of possible outbreaks and other health-related events. The system was implemented at four sites were mass gathering events occurred over the three-day visit. Data were collected by administering a simple questionnaire on a tablet, which collected information about sociodemographics, syndromic diagnoses, and outcomes of the patients that sought medical care. Additionally, a descriptive epidemiological assessment was performed during the event. A total of 150 individuals were attended at the designated places during the event. Of these, 56.7% were female and 90.7% aged > 15 years. The majority of the patients (74.7%) sought care on the third day of the event, which was held at the Zimpeto National Stadium. The most common diagnoses were hypertension (20.7%), hypothermia (15.3%), and headache (11.3%). Almost all cases (95.0%) were discharged, (4.0%) cases were transferred and (1.0%) case resulted in death on the way to the health facility. The surveillance system strategy developed to detect real-time public health events during the Pope?s visit was successfully implemented. No outbreak was identified during the event.

## Introduction

Mass gathering events are preplanned collective activities that may be of a cultural, sporting, commercial, religious, social or political nature, held at a pre-determined time, which attract a large contingent of people and may originate in the host country or other parts of the world. The event duration can range from hours to days [1-3]. By their nature, such events can facilitate the transmission of infectious diseases [1-4] and exacerbate symptoms of chronic disease [5]. Due to the large influx of people and attendee movement, these events can also impose a substantial burden on the local health system [1,3]. The high concentration of host country nationals and foreigners, with different habits and customs, represents a risk for the introduction and widespread transmission of disease [2,3,6,7]. From September 4-6, 2019, Mozambique hosted Pope Francis, the supreme head of the Roman Catholic Church. Worship services and meetings were held at various locations through the capital city during his visit. The Mozambican Ministry of Health (MoH) implemented a real-time surveillance system to monitor and detect any outbreaks or public health emergencies that might occur during Pope's visit to Mozambique and to enable prompt public heath intervention.

## Methods

A daily syndromic surveillance system was implemented in mobile health units located in the four places were Pope Francis held public meetings during his visit to Maputo City: Maputo International Airport (first day); Maxaquene Pavilion and the Cathedral of the Immaculate Conception (second day); and Zimpeto Stadium (third day), which is the largest sports arena in the country, with a capacity of 42,000 people. Data were collected in real-time using tablets to administer a structured short form survey to individuals seeking care. The tool was piloted beforehand to allow for corrections and evaluation of its feasibility. Prior to data collection, surveillance health staff received a short training on survey administration. Data were entered and analyzed using the ODK package. A cross-sectional descriptive epidemiological assessment was performed, using measures of frequency and distribution of the reported symptoms and diagnoses, over the three-day period, disaggregated by age, gender, and nationality. A daily report was issued and shared with the stakeholders.

## Results

**Figure 1:**
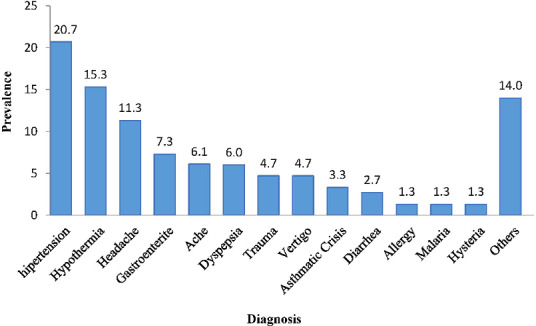
Distribution of diagnoses recorded during the papal visit, Mozambique, 2019

During the period of Pope Francis' visit to Mozambique, a total of 150 individuals sought medical care at the four health posts, of which 85 (56.7%) were female and 136 (90.7%) aged > 15 years. The event at which the majority (74.7%) of individuals sought care occurred on the third day at Zimpeto National Stadium. Of the patients who presented for medical care during the three-day visit to Mozambique, most were female (56.7%), and > 15 years of age (87.5%). The mean age of individuals seeking care was 39 years and ranged from 8 to 99 years (Table 1). From the records the most common diagnoses were hypertension (HTN) (20.7%), hypothermia (n = 23, 15.3%), headache (n = 17, 11.3%) and gastroenteritis (n = 11, 7.3%). There were also 14 cases (9.3%) of illness that could not be combined with another category (Figure 1). Regarding the outcome of the patients, 143 (95.0%) were discharged, six cases were transferred to José Macamo General Hospital, a referral health facility located in Maputo City. One death, a 49-year-old female, occurred on the way to the health unit. the The surveillance team could not obtain the cause of death, however, the medical team noted that the patient did not present with any infectious disease symptoms.

**Table 1:** General characteristics of patients who sought health consultations during the papal visit, Mozambique, 2019

Characteristics	Day 1		Day 2		Day 3			
	Maputo International Airport (n = 2)		Maxaquene Pavilion e Cathedral (n = 36)		National Stadium of Zimpeto (n =112)		Total	
Characteristics	n	%	n	%	n	%	N	%
Sex								
Male	1	50.0	20	55.6	44	39.3	65	43.3
Female	1	50.0	16	44.4	68	60.7	85	56.7
Age range (Years)								
Mean (range)	39 (8,99)							
0-4	0	0	0	0	0	0	0	0
5-14	0	0	0	0	14	12.5	14	9.3
>=15	2	100	36	100	98	87.5	136	90.7
Nationality								
Mozambican	2	100	36	22.4	100	89.3	138	92.0
South African	0	0	0	0	12	10.7	12	8.0

## Discussion

The most well-attended event of the papal visit, a public mass, occurred on the last day at Zimpeto National Stadium. Similar results were found in other mass events, in which the peak days of the visits were in the days with the highest concentration of people [8, 9]. There was a greater demand for care among adults and females. The greater proportion of women seeking medical care may be related to the fact that women seek health services more than men [10], and women tend to attend more religious events than men [11, 12]. Most patients presented with HTN, hypothermia, and abdominal pain. HTN was the most frequent diagnosis during this mass gathering and during the Mozambique IX National Festival of Culture in Sofala Province in 2016, where out of 355 patients examined, 31 (20.3%) were diagnosed with HTN [9]. Attending a mass gathering event may entail extended periods of waiting and or walking, thus resulting in fatigue and stress, thereby resulting in increased HTN.

This result may be associated with the high level of stress that these events may cause due to the large conglomeration of people, long periods of waiting, walking long distances, fatigue and other factors [3, 7, 9, 13]. Hypothermia was one of the most frequent diagnoses on the last day of the event, which had the greatest number of attendees. Hypothermia is a common diagnosis during this type of event [14]. When the mass gatherings events are held in stadiums or open spaces, temperature, humidity, and degree of mobility of attendees influence the patient presentation [15]. During that day, there was a marked reduction in temperature and drizzle in an open and uncovered environment. Many participants were not properly dressed, which may have contributed to this diagnosis. The majority of individuals with disease had mild symptoms, consistent with similar mass gathering events held in the country [9]. Some limitations need to be considered. The total estimated population that attended each event was not known, thus the prevalence of syndromes and diagnoses could not be estimated. Since this analysis was limited to fixed health sites established at the four locations, the results can be generalized to all health facilities that provided basic medical services during the papal visit. It was not possible to determine the cause of death. Detailed data on risk factors during the events were not collected. The system was implemented only during the events days, in this way the system may have lost some cases that showed symptoms before the events.

## Conclusion

As part of the preparedness and response plan, public health authorities, in collaboration with event organizers, need to enhance their communicable disease surveillance systems, based on the nature of the event and expected attendees, to enable early detection of potential public health threats. The real-time surveillance system set up during Pope Francis' visit to Mozambique proved to be effective in monitoring the symptoms experienced by attendees who sought medical care; symptoms were generally of moderate severity. There was more demand for health services from adult women and during the event at National Zimpeto Stadium, which was characterized by a sharp drop in temperature and rainfall, and several cases of hypothermia were detected. Pre-event planning for mass gathering events must consider a wide range of public health risks, including extreme weather. Furthermore, strengthened health education to educate attendees on health-related risks during mass gathering events could prevent potential disease transmission.

### What is known about this topic

- Real-time events have the potential to import and export infectious diseases;
- Preparation for mass gatherings events needs to include public health measure in advance;
- Real-time surveillance systems during mass gathering events help to improve real-time reporting and rapidly detect health threats.

### What this study adds

- It is important to develop appropriate health promotion and prevention education messages and tools for attendees in mass gathering events;
- Pre-event planning for mass gathering events in open spaces must consider a wide range of public health risks, including weather and environmental conditions;
- Surveillance systems for mass gathering events should be implemented during the days prior to and immediately after the event.

## Competing interests

The authors declare no competing interests.
